# Elevated muscle pain induced by a hypertonic saline injection reduces power output independent of physiological changes during fixed perceived effort cycling

**DOI:** 10.1152/japplphysiol.00325.2023

**Published:** 2024-05-30

**Authors:** Callum A. O’Malley, Ryan Norbury, Samuel A. Smith, Christopher L. Fullerton, Alexis R. Mauger

**Affiliations:** ^1^School of Sport and Exercise Sciences, University of Kent, Canterbury, United Kingdom; ^2^School of Sport and Health Sciences, University of Exeter, Exeter, United Kingdom; ^3^Faculty of Sport, Technology, and Health Sciences, St Mary’s University Twickenham, London, United Kingdom; ^4^Faculty of Health Sciences and Sport, University of Stirling, Stirling, United Kingdom

**Keywords:** effort, exercise behavior, muscle pain, psychophysiology, self-regulation

## Abstract

Pain is a naturally occurring phenomenon that consistently inhibits exercise performance by imposing unconscious, neurophysiological alterations (e.g., corticospinal changes) as well as conscious, psychophysiological pressures (e.g., shared effort demands). Although several studies indicate that pain would elicit lower task outputs for a set intensity of perceived effort, no study has tested this. Therefore, this study investigated the impact of elevated muscle pain through a hypertonic saline injection on the power output, psychophysiological, cerebral oxygenation, and perceptual changes during fixed perceived effort exercise. Ten participants completed three visits (1 familiarization + 2 fixed perceived effort trials). Fixed perceived effort cycling corresponded to 15% above gas exchange threshold (GET) [mean rating of perceived effort (RPE) = 15 “hard”]. Before the 30-min fixed perceived effort exercise, participants received a randomized bilateral hypertonic or isotonic saline injection in the vastus lateralis. Power output, cardiorespiratory, cerebral oxygenation, and perceptual markers (e.g., affective valence) were recorded during exercise. Linear mixed-model regression assessed the condition and time effects and condition × time interactions. Significant condition effects showed that power output was significantly lower during hypertonic conditions [*t*_107_ = 208, *P* = 0.040, β = 4.77 W, 95% confidence interval (95% CI) [0.27 to 9.26 W]]. Meanwhile, all physiological variables (e.g., heart rate, oxygen uptake, minute ventilation) demonstrated no significant condition effects. Condition effects were observed for deoxyhemoglobin changes from baseline (*t*_107_ = −3.29, *P* = 0.001, β = −1.50 ΔμM, 95% CI [−2.40 to −0.61 ΔμM]) and affective valence (*t*_127_ = 6.12, *P* = 0.001, β = 0.93, 95% CI [0.63 to 1.23]). Results infer that pain impacts the self-regulation of fixed perceived effort exercise, as differences in power output mainly occurred when pain ratings were higher after hypertonic versus isotonic saline administration.

**NEW & NOTEWORTHY** This study identifies that elevated muscle pain through a hypertonic saline injection causes significantly lower power output when pain is experienced but does not seem to affect exercise behavior in a residual manner. Results provide some evidence that pain operates on a psychophysiological level to alter the self-regulation of exercise behavior due to differences between conditions in cerebral deoxyhemoglobin and other perceptual parameters.

## Graphical abstract

**Figure:**
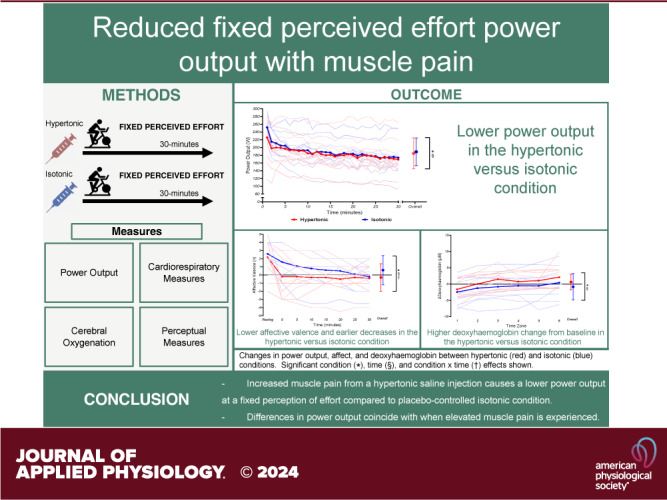

## INTRODUCTION

Effort-based decision-making is central to task performance (1). Ultimately, individuals will enact a behavior if the subjective evaluation of the potential reward meets/exceeds the effort to obtain the outcome (2). Naturally, exercise imposes a catalog of new sensory and perceptual experiences (3) that impact the perceived value of a task (2, 4). Consequently, it becomes important for individuals to self-regulate their behavior and psychophysiological state to promote a continued investment of effort (5).

Muscle pain is a perception arising from the integration of nociceptive stimulations of type III and IV muscle afferents (6). Notably, pain has been observed to consistently inhibit exercise performance (3, 7–12). On one hand, the nociceptive element tends to impose numerous inhibitive, neurophysiological alterations along the corticospinal pathways (13, 14). For instance, Martinez-Valdes et al. (15) identified that during conditions with higher nociception, the recruitment threshold of fatigue-prone, fast-twitch fibers was lowered whereas fatigue-resistant, slow-twitch fibers saw reduced firing rates. Concomitantly, numerous studies demonstrate that experimental methods that increase nociception/pain (e.g., hypertonic saline, ischemia, electrical, and/or thermal stimulation) cause an increase in corticospinal inhibition as well as a decrease in corticospinal excitability (13–17). Thus, the underlying nociceptive aspect to pain elicits a compensatory increase in central drive to maintain an exercise intensity compared to conditions with less/lower nociceptive stimulation (10, 11), thereby increasing perceptions of effort for a set intensity of exercise (12, 18).

On the other hand, pain also inflicts conscious, psychophysiological changes (19). To illustrate, pain has evidenced a marked impact on the hedonic (e.g., less pleasurable) and motivational (e.g., less willing to apply effort) aspects of the affective experience, causing people to feel and perform worse when in pain (20). Subsequent data from neurophysiological studies indicate an increased activation of cortical areas associated with inhibitory control (21), particularly when performing with a negative affective valence due to pain (1, 19, 20). In turn, continued engagement in inhibitory control is believed to exact a motivationally fatiguing effect (22) as well as being associated with a subjective feeling of effort (1). Therefore, it is unsurprising that during painful tasks that require inhibitory control, a given exercise intensity feels more effortful (1, 18).

Collectively, past studies imply that pain and its underlying nociceptive component tend to have negative psychophysiological effects (19) as well as a net inhibitive effect on corticospinal transmission of central drive (13, 14). Therefore, for a fixed task intensity like a time-to-exhaustion trial, a compensatory increase in central drive is required to maintain the intensity, causing a higher perception of effort for a given intensity (18). Alternatively, when the task paradigm is flipped to a fixed perceived effort task, pain conditions would be expected to cause a reduced intensity/workload compared with nonpainful conditions. However, no study has tested this yet. Moreover, as pain is a compelling sensory and emotional experience that must be endured when undertaking exercise (23), it is important to understand the methods that individuals use to self-regulate and cope with pain without compromising exercise performance (5, 23).

Therefore, the aims of this study were twofold. Primarily, the present study aimed to investigate the impact of elevated pain perceptions through a hypertonic saline injection on power output and psychophysiological state during a fixed perceived effort task. Second, the present study also aimed to investigate the self-regulatory responses [i.e., changes in power output (behavioral) and cerebral hemodynamics (cognitive) as indicators of the self-regulatory strategies] that were used to maintain a fixed perceived effort during hypertonic (painful) or isotonic (placebo-control) conditions.

It was hypothesized that mean power output would be lower in the hypertonic versus isotonic condition (condition effect). Second, it was hypothesized that the decreases over time in power output would be steeper in the hypertonic versus isotonic condition (condition × time interactions). It was also hypothesized that changes in cerebral oxygenation markers from baseline would be greater in the pain versus isotonic condition, indicating more inhibitive control (24, 25). Finally, a series of secondary hypotheses were made that markers of physiological strain (e.g., heart rate, ventilatory parameters, blood lactate) would be lower in the hypertonic than in the isotonic condition, whereas perceptual markers like affective valence would be lower in the hypertonic versus isotonic condition.

## MATERIALS AND METHODS

### Participants

Ten healthy and recreationally trained cyclists (2 female) with mean ± SD age 28.9 ± 6.6 yr, height 175.8 ± 6.1 cm, mass 72.1 ± 8.0 kg, physical activity 6.1 ± 2.9 h·wk^−1^, and maximum relative oxygen uptake (V̇o_2_·kg^−1^) 52.6 ± 7.2 mL·kg^−1^·min^−1^ volunteered to participate in this study. An a priori calculation using an effect size (*d_z_* = 1.09) from Ref. 11 that used an identical saline injection procedure, α = 0.05, and β = 0.8, determined a required sample size of 10 to determine a sufficient effect on power output during a fixed perceived effort trial with an actual β = 0.82. All participants reported at least 3 yr of cycling experience, current engagement in cycling activity, and an “excellent” maximum oxygen uptake (V̇o_2max_) according to Ref. 26 to qualify for this study. All participants were free from any musculoskeletal injuries in the previous 6 mo, with no cardiovascular disease, neurological disorders, or blood-borne viruses, and participants did not use dietary supplements or medication throughout the entire study. Before all data collection sessions, participants abstained from food (2 h), caffeine (4 h), analgesics (8 h), and alcohol (48 h) and refrained from vigorous exercise (48 h). Female participants reported being eumenorrheic and were scheduled so that all visits were conducted within the same stage of menses (luteal phase). All participants provided written informed consent before testing for this School of Sport and Exercise Sciences Research Ethics Advisory Group-approved study (Prop #11_20_21) which was conducted according to the scientific principles outlined within the Declaration of Helsinki.

### Procedures

**Figure 1.**
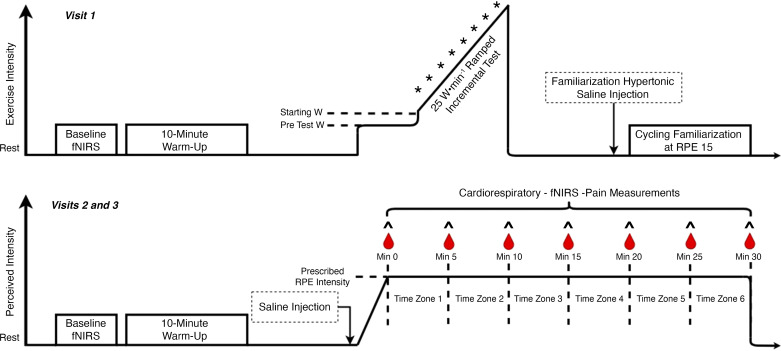
Visual representation of study protocols. W represents power output; ∧ indicates affective valence and self-efficacy measurements; 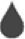 represents blood lactate measurements; * represents rating of perceived effort (RPE) measurements. fNIRS, functional near-infrared spectroscopy measures.

The present study implemented a randomized, single-blinded, within-subject design whereby the lead researcher was blinded to which conditions were being completed. Initially, the researchers aimed to complete a double-blinded design; however, the infusion of hypertonic saline may naturally be distinguished from that of isotonic saline by participants (7, 9–12). On three separate occasions (Fig. 1), participants were required to visit the same laboratory. Each visit was conducted at the same time of day (±2 h) in similar ambient environments (mean ± SD temperature 19.6 ± 3.8°C, humidity 51.9 ± 8.4%, barometric pressure 751.9 ± 7.7 mmHg). Each visit was separated by a minimum of 3 days and a maximum of 7 days.

At the start of each session, participants’ anthropometrics were recorded, and they were provided with a full brief of the procedures, equipment, and perceptual scales. Participants were fitted to the functional near-infrared spectroscopy (fNIRS) device (PortaLite MK II; Artinis Medical Systems BV, Arnhem, The Netherlands) and asked to sit completely still for 5 min during baseline measurements. Participants were also fitted with a heart rate monitor (ANT+; Cyclus 2, Leipzig, Germany) to assess heart rate on a beat-by-beat basis and provided a 20-μL resting blood lactate sample from the right index finger to be assessed with an automated lactate analyzer (Biosen: C-Line; EKF Diagnostics, GmbH, Barlaben, Germany). Finally, participants provided baseline values for each perceptual scale (see *Perceptual Scales*).

Participants performed identical 10-min warm-ups at a rating of perceived effort (RPE) of 11 (“light”) on the cycle ergometer (Cyclus 2). After the warm-up, participants were afforded 5 min of passive recovery before remounting the cycle ergometer to begin the respective exercise tasks for each session. During all exercise tasks, participants were fitted to a calibrated gas analyzer system (Cortex Metalyzer model 3B; Leipzig, Germany) to assess pulmonary ventilation [e.g., V̇o_2_·kg^−1^, minute ventilation (V̇e), and breathing frequency] on a breath-by-breath basis. After exercise, participants completed a short questionnaire pack; on completion, they were debriefed and exited the laboratory.

### *Visit 1*: Ramped Incremental Test and Familiarization

The first visit consisted of a ramped incremental test and a familiarization to fixed perceived effort cycling with bilateral hypertonic saline administration. The ramped incremental test involved an initial 3-min stabilization period at 80% starting intensity (males = 80 W, females = 40 W). Participants were asked to cycle at a comfortable cadence ∼80 revolutions·min^−1^ and were recommended to gradually increase cadence over the course of the test. The incremental ramped test began at 100 W (males) or 50 W (females) with 25 W·min^−1^ increments. These intensities were selected according to pilot test data to ensure that ramped incremental tests lasted between 8 and 12 min, as previously recommended (27).

During the ramped incremental tests, breath-by-breath analysis of oxygen consumption (V̇o_2_), carbon dioxide expulsion (V̇co_2_), V̇e, and breathing frequency were taken. An RPE response was obtained at each minute (including starting intensity and at the point of exhaustion). Finally, a blood lactate sample was taken at the point of exhaustion. Cerebral oxygenation via fNIRS, affective valence, and pain intensity were not measured during the ramped incremental test. Task cessation demarcated when the participant believed they reached volitional exhaustion or cadence fell below 60 revolutions·min^−1^ for >5 s despite strong verbal encouragement.

After the ramped incremental test, participants received 15-min passive recovery and were then prepared for a 10-min fixed perceived effort cycle at RPE 15 (“hard”) after receiving a bilateral hypertonic saline intramuscular injection for familiarization. A full explanation of the fixed perceived effort trials can be seen in *Visits 2 and 3: Fixed Perceived Effort Trials*.

### Determination of Fixed Perceived Effort Intensity in *Visits 2* and *3*

With the V̇-slope method (28), gas exchange threshold (GET) was matched to the point at which V̇o_2_ values above and below the breakpoint of V̇co_2_ diverged from the intersection of the two linear regression lines. Secondary criteria including ventilatory equivalents (first divergence of ventilatory equivalent of oxygen and carbon dioxide), end-tidal volumes (first divergence of end-tidal volumes for oxygen and carbon dioxide), respiratory exchange ratio (reaching a value of 1.00), and a secondary researcher confirmed GET identification (26). Once GET was determined, V̇o_2_ values 15% above GET (GET_+15%_) were calculated. Plotting GET_+15%_ V̇o_2_ against power output from the ramped incremental test, a regression equation (*y* = *mx* + *c*) derived what power output corresponded to the GET_+15%_ V̇o_2_. Finally, power output data were plotted against ramped incremental RPE responses in which a similar regression equation was used to identify RPE (RPE_+15%GET_) at the corresponding power output at GET_+15%_. This RPE was rounded to the nearest whole number and used as the RPE reference for subsequent fixed perceived effort cycling in *visits 2* and *3* [mean ± SD RPE_+15%GET_ = 14.7 ± 0.4, 8*n* = RPE 15 (“hard”), 2*n* = RPE 14 (between “somewhat hard” and “hard”)].

### *Visits 2* and *3*: Fixed Perceived Effort Trials

Both experimental sessions were single-blinded and randomized. After the same preparation, baseline, and warm-up protocols as *visit 1*, participants were prepared to receive two simultaneous, bilateral saline injections before commencing a 30-min fixed perceived effort cycle. Injections involved a bolus of 1 mL of saline (hypertonic = 5.85% NaCl, isotonic = 0.9% NaCl) injected into the middle third of the muscle belly of the vastus lateralis on each leg. Injection sites were measured and marked to ensure consistent locality of injection. Sites were cleaned with an alcoholic swab, and saline was manually infused with a 3-mL Luer-Lok syringe (BD, New Jersey) connected to a 3.8-cm 25-gauge hypodermic needle (SurGuard2; Terumo, Japan) over a 20-s window (insertion, 5-s pause, 10-s infusion period, 5-s pause, withdrawal). A hypertonic saline model was utilized because several studies have validated its ability to mimic exercise-induced pain experiences across different physical task modalities (9–11, 16, 17, 29, 30) as well as demonstrating its replicability (29). However, it does present some difficulties with blinding as participants can distinguish which condition they are completing, which may generate some confounding effects on behavioral, motivation (1, 19), and other psychophysiological indexes such as hyperventilation (29).

Immediately after the injection procedure, participants began cycling and ramped up to the required RPE (mean ± SD time to begin fixed perceived effort task: hypertonic = 27 ± 9 s, isotonic = 29 ± 9 s). After this, the fixed perceived effort trial commenced. During this, power output, heart rate, gas parameters, cerebral oxygenation parameters via fNIRS, and pain measurements were assessed continually and affective valence and blood lactate were assessed every 5 min.

Crucially, the task was a fixed perceived effort trial (see Ref. 31); therefore, throughout the trial, participants were blinded from all performance-related variables (e.g., power output, time on task) except for cadence. In this way, participants’ sole focus was to maintain a fixed perceived effort. Participants were asked to maintain a cadence between 80 and 90 (±2) revolutions·min^−1^ that was replicated across both sessions (mean ± SD 86 ± 3 revolutions·min^−1^). However, power output could be changed at any point throughout the exercise to maintain the fixed perceived effort with virtual gears on the Cyclus 2 ergometer console that changed the resistance at the set cadence. The researcher provided a reminder of the RPE definition (32) and the need for the participant to be at a fixed perceived effort every 2 min.

### fNIRS Measurement

Cerebral oxygenation was assessed through a portable fNIRS device. The device was placed on the surface of the forehead aligned with the left prefrontal cortex between Fp1 and F3 (International EEG 10-20 System), as this aligns with relevant cerebral centers for executive motor control (33). Before application, the skin was wiped with an alcohol swab and a thin transparent film was placed over the site to prevent any sweat interfering with the device. To protect from light interference, a black bandana was placed over the device, which held it stationary. Furthermore, the wire leading from the optode to the laptop was taped tightly onto the cycle ergometer and adjoining table to avoid movement artifacts. Precalibration adjusted an age-dependent differential path-length factor, and data were sampled at 10 Hz from six optodes at wavelengths between 760 and 850 nm according to manufacturer’s guidelines. Data were sampled from single, long-separation channels. Moreover, according with the manufacturer’s guidelines and prior studies (34), a low-pass filter of 0.1 Hz was applied to all participant data and a visual inspection of all data was completed to identify and remove any movement artifacts present in the data. A 5-min resting baseline was completed at the beginning of each session, whereby any fNIRS data obtained during subsequent exercise tasks were represented as changes from baseline (Δ) (35). Therefore, fNIRS data during exercise were expressed as change in oxyhemoglobin (ΔO_2_Hb), deoxyhemoglobin (ΔHHb), total hemoglobin (ΔtHb), and tissue saturation index (TSI = ΔO_2_Hb/ΔtHb × 100) compared to resting baseline, with an arbitrary average baseline value denoting 0 μM, in accordance with previous research (36, 37).

### Perceptual Scales

#### RPE scale.

The 15-point Borg RPE scale (38) denoted “How hard, heavy, and strenuous does the exercise consciously feel to drive the working muscles and for your breathing?” (31). Responses ranged from 6 (“no effort,” “like when you were sat during the fNIRS baseline doing absolutely nothing”) to 20 (“maximum effort,” “like giving everything you have got like at the end of a V̇o_2max_ test”). Appropriate anchors were given before exercising to facilitate the consistency of participant responses (39, 40).

#### Affective valence scale.

The feeling scale (41) denoted “How are you feeling at the present moment of the exercise?”. Responses ranged on an 11-point Likert scale from +5 (“I feel very good”) to −5 (“I feel very bad”), with a middle value of 0 denoting “neutral.”

#### Pain measurement.

During experimental exercise trials, a continual rating of exercise-induced pain intensity was obtained by participants using a moveable cursor on an electronic visual analog scale (VAS) that sampled a recording every 5 s. Responses ranged from 0 = “no pain” to 100 = “worst imaginable pain” (8). This device was placed on the handlebars of the ergometer for ease. Participants were instructed to anchor the uppermost pain rating to the worst exercise-induced pain they had previously experienced (8, 42).

Pain quality was assessed by using the long-form McGill Pain Questionnaire (43) to assess several pain elements such as sensory, affective, and evaluative qualities. Therefore, the McGill Pain Questionnaire allows a more multidimensional consideration of pain that goes beyond the simple magnitude of pain. Each category contains adjectives that are ranked in ascending order according to implied pain intensity (e.g., descriptor 1 assigned a value of 1). A subclass rating index denoted a sum for each subclass, and a total pain rating index denoted a sum of all subclasses. The McGill Pain Questionnaire was administered after each fixed perceived effort exercise task, where participants were required to select one word from each subcategory if any of the descriptors applied.

### Analysis

Power output data were averaged across each minute of the 30-min fixed perceived effort trials. All other continuous data [e.g., physiological (except blood lactate), cerebral oxygenation markers] and pain intensity ratings were averaged across six 5-min time zones (e.g., *time zone 1* = minute 00:00–04:59). Affective valence and blood lactate were analyzed according to the minute they were extracted (e.g., *minutes 0*, *5*, etc.).

All data were exported to Jamovi (v 2.3; JAMOVI, Sydney, Australia) and were assessed for normality and symmetry with Q-Q plots and a Shapiro–Wilk test before any further analysis. Any data that exceeded 2 SDs from the group mean were excluded from further analysis, although subsequent analysis evidenced that no participant’s data exceeded 2 SDs from the group mean. A series of paired-samples *t* tests were conducted to assess differences between conditions in resting responses for perceptual markers and blood lactate.

A random-intercepts linear mixed-effects model regression was conducted to assess the condition and/or time effects as well as the condition × time interactions on all dependent variable data. Condition effects observed differences between hypertonic and isotonic (placebo-control) conditions. Time effects observed differences over the course of the 30-min perceived effort task. Condition × time interactions observed the differences between conditions in changes to a set variable over time. The generalized form for the linear mixed-model regression is presented below (*Eq. 1*), showing that the grouping/cluster variable was each participant.

(*1*) (Dependent Variable) = Condition + Time Zone + Condition:Time Zone + (1|Participant)

The variables of condition and time were set as fixed effects. Models were fitted according to the group intercept. Results from the linear mixed-model regression were reported as *t* values, as time was entered as a continuous variable. Another benefit to this method is that reporting of estimated marginal means (β-coefficient) denotes the raw mean differences between the two conditions as an effect size with supplementary 95% confidence intervals (95% CIs). A normality test was conducted on the residual values, and if they violated normality a Wilcoxon signed-rank test was reported with a rank biserial correlation (*r*) denoting effect size. All data reported for the mixed-model regression are according to isotonic-hypertonic comparisons, with positive *t* and β values showing a higher value in the isotonic versus hypertonic condition.

Data from the McGill Pain Questionnaire underwent a basic frequency analysis whereby each descriptor was assigned a score (1–5) according to its severity. Each of the 20 categories of descriptors was grouped according to their subclass, and a total score for each subclass was calculated for each condition and participant. Next, all subclass totals were calculated to also create a total pain rating index across each condition and participant. Mean scores across the cohort for each subclass as well as the total pain rating index underwent a series of *t* tests to assess the differences between conditions. For clarity, only descriptors that were selected by over one-third of the cohort are presented in Table 1. A Wilcoxon signed-rank rest was reported if data violated normality, and a Cohen’s *d* was reported to denote effect size. The alpha level for all tests was set at *P ≤* 0.05.

**Table 1.** Frequency of descriptors selected and subclass scores for pain quality

Subclass		Hypertonic	Isotonic
Sensory		Hot (40%) Sharp (50%) Tender (60%) Burning (40%) Throbbing (50%) Tugging (50%)	Hot (60%) Sharp (50%) Tender (60%) Pricking (40%) Dull (40%) Aching (40%) Pulling (50%) Tingling (50%) Pressing (60%)
	SRI	17 ± 5	14 ± 6#
Affective		Grueling (40%) Tiring (70%) Sickening (40%) Fearful (40%) Wretched (40%)	Grueling (40%) Tiring (70%)
	SRI	5 ± 3	3 ± 2‡
Evaluative		Intense (60%)	Annoying (40%)
	SRI	3 ± 1	2 ± 2*#
Miscellaneous		Tight (40%) Radiating (40%)	Tight (80%) Spreading (40%) Nagging (50%)
	SRI	5 ± 2	4 ± 2*#
	PRI (T)	30 ± 8	22 ± 11*‡

Subclass rating index (SRI) and pain rating index total [PRI (T)] scores presented as means ± SD. *Significant difference between conditions. #Moderate effect size; ‡large effect size.

## RESULTS

### Standardization

Before beginning the experimental fixed perceived effort cycling trials, all participants rated no pain (0), and blood lactate was not significantly different between conditions (hypertonic = 1.53 m·mol^−1^ versus isotonic = 1.45 m·mol^−1^, *P* = 0.327, *d* = 0.18). In addition, affective valence did not differ between conditions before exercise (hypertonic = 2.2 vs. isotonic = 2.6, *P* = 0.111, *d* = 0.21).

### Power Output and Physiological Markers

**Figure 2.**
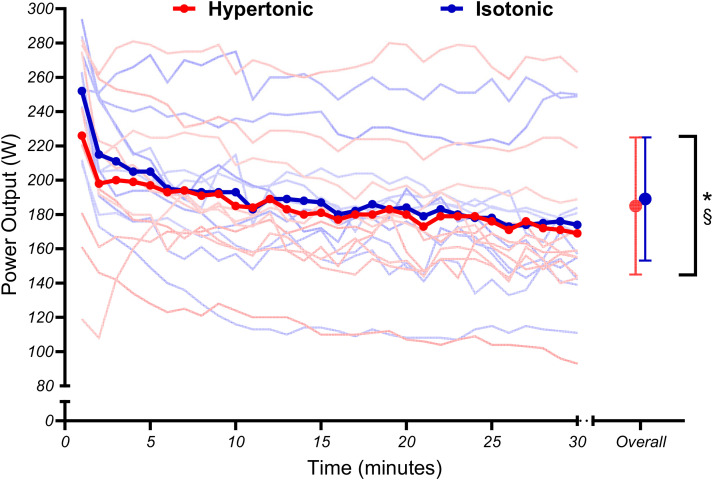
Mean group (thick line) and individual (thin lines) power output data during fixed perceived effort trials. Significant condition (*) and time (§) effects are illustrated. Error bars denote SDs from the mean.

Power output was found to be significantly lower in the hypertonic compared to isotonic condition, with significant main effects for condition (*t*_107_ = 2.08, *P* = 0.040, β = 4.77 W [0.27,9.26]) being observed. Power output also decreased over time in both conditions, with main effects for time (*t*_107_ = −6.11, *P* = 0.001, β = −5.80 W [−7.66,3.94]) being observed (Fig. 2). The trajectories of power output changes did not significantly differ between conditions, as there was no condition × time interaction (*t*_107_ = −1.32, *P* = 0.189, β = −1.78 [−4.41,0.86]).

**Figure 3.**
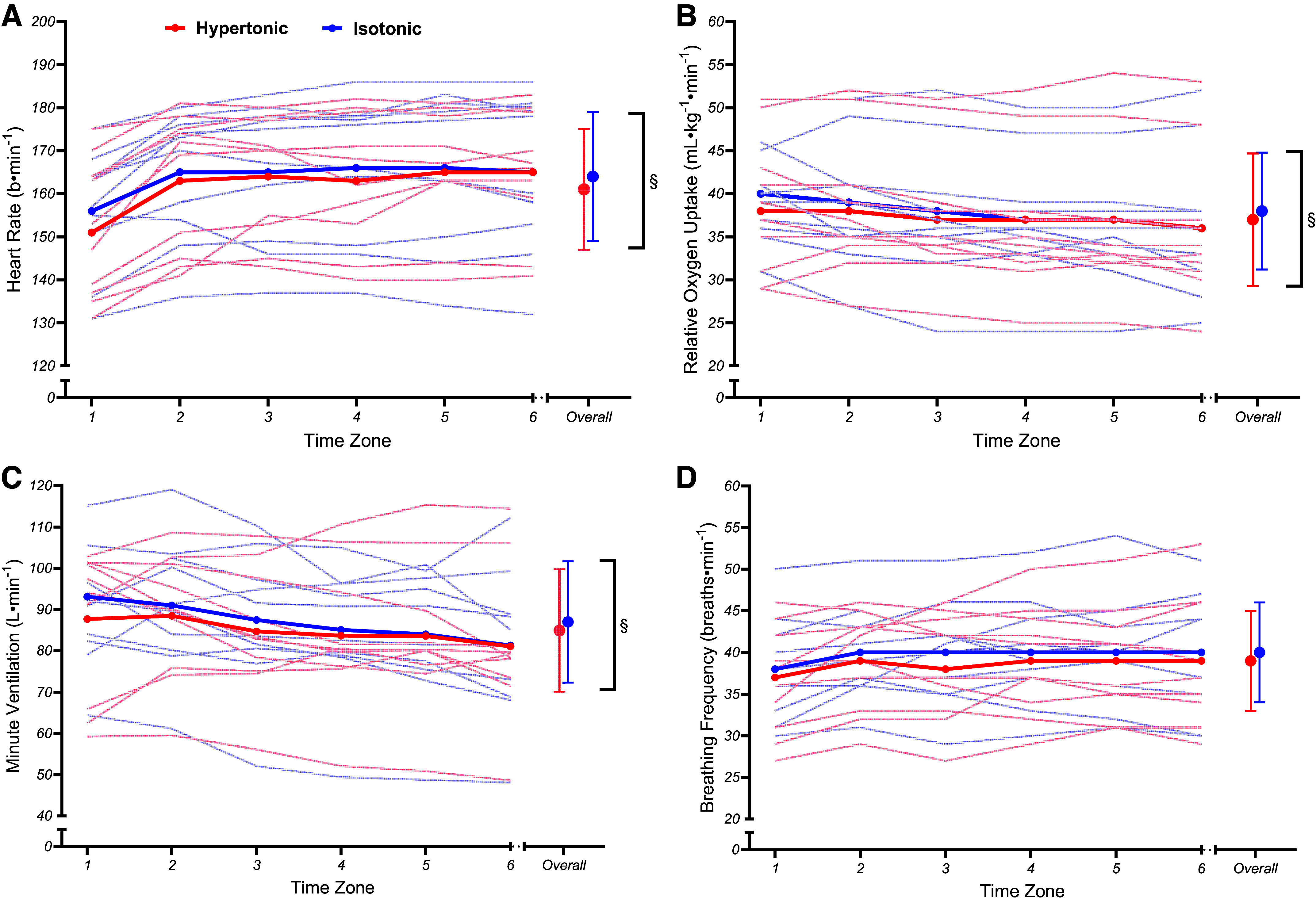
Mean group (thick line) and individual (thin lines) heart rate [beats (b)·min^−1^; *A*], relative oxygen uptake (V̇o_2_·kg^−1^; *B*), minute ventilation (V̇e; *C*), and breathing frequency (*D*) cardiorespiratory data during fixed perceived effort trials. Significant time (§) effects are illustrated. Error bars denote SDs from the mean.

There were no differences in heart rate between conditions (*t*_107_ = 1.69, *P* = 0.094, β = 1.82 beats·min^−1^ [−0.29,3.92]). However, heart rate did increase across both conditions, as a significant main effect for time (*t*_107_ = 5.63, *P* = 0.001, β = 1.77 beats·min^−1^ [1.15,2.39]) was observed (Fig. 3*A*). Trajectories in heart rate changes did not differ between conditions (*t*_107_ = −1.17, *P* = 0.246, β = −0.73 [−1.97,0.50]).

Similarly, V̇o_2_·kg^−1^ (*t*_107_ = 1.34, *P* = 0.182, β = 0.57 mL·min^−1^·kg^−1^ [−0.26,1.39]) and V̇e (*t*_107_ = 1.43, *P* = 0.157, β = 2.12 L·min^−1^ [−0.79,5.04]) did not demonstrate a significant condition effect. However, V̇o_2_·kg^−1^ (*t*_107_ = −5.29, *P* = 0.001, β = −0.65 mL·min^−1^·kg^−1^ [−0.90,−0.41]) and V̇e (*t*_107_ = −4.31, *P* = 0.001, β = −1.88 L·min^−1^ [−2.73,−1.02]) did demonstrate significant changes in values over time (Fig. 3, *B* and *C*). No significant condition × time interactions were observed for V̇o_2_·kg^−1^ (*t*_107_ = −0.86, *P* = 0.394, β = −0.21 [−0.70,0.27]) or V̇e (*t*_107_ = −1.10, *P* = 0.273, β = −0.96 [−2.67,0.75]).

**Figure 4.**
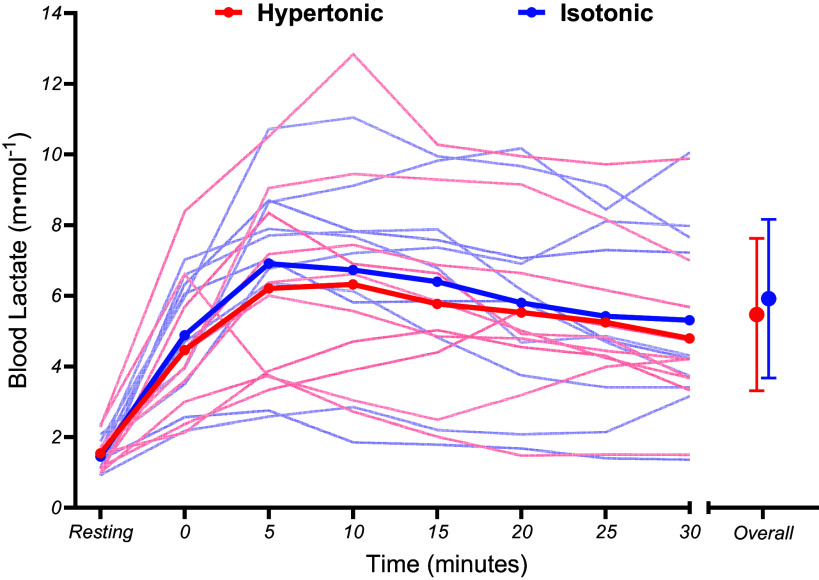
Mean group (thick line) and individual (thin lines) blood lactate responses during fixed perceived effort exercise. Error bars denote SDs from the mean.

Breathing frequency was not significantly different between conditions (*t*_107_ = 1.72, *P* = 0.088, β = 1.00 breaths·min^−1^ [−0.14,2.14]) and did not differ over time (*t*_107_ = 1.82, *P* = 0.072, β = 0.31 breaths·min^−1^ [−0.02,0.64]) (Fig. 3*D*). In addition, breathing frequency did not show a significant condition × time interaction (*t*_107_ = −0.32, *P* = 0.750, β = −0.11 [−0.77,0.56]). Finally, no significant main effects for condition (*t*_127_ = 1.84, *P* = 0.068, β = 0.45 m·mol^−1^ [−0.03,0.92]) or time (*t*_127_ = −1.29, *P* = 0.200, β = −0.02 m·mol^−1^ [−0.04,0.01]), were observed for blood lactate. In addition, condition × time interactions for blood lactate (*t*_127_ = −0.27, *P* = 0.789, β = −0.01 [−0.05,0.04]) were insignificant (Fig. 4).

### Cerebral Oxygenation Markers

**Figure 5.**
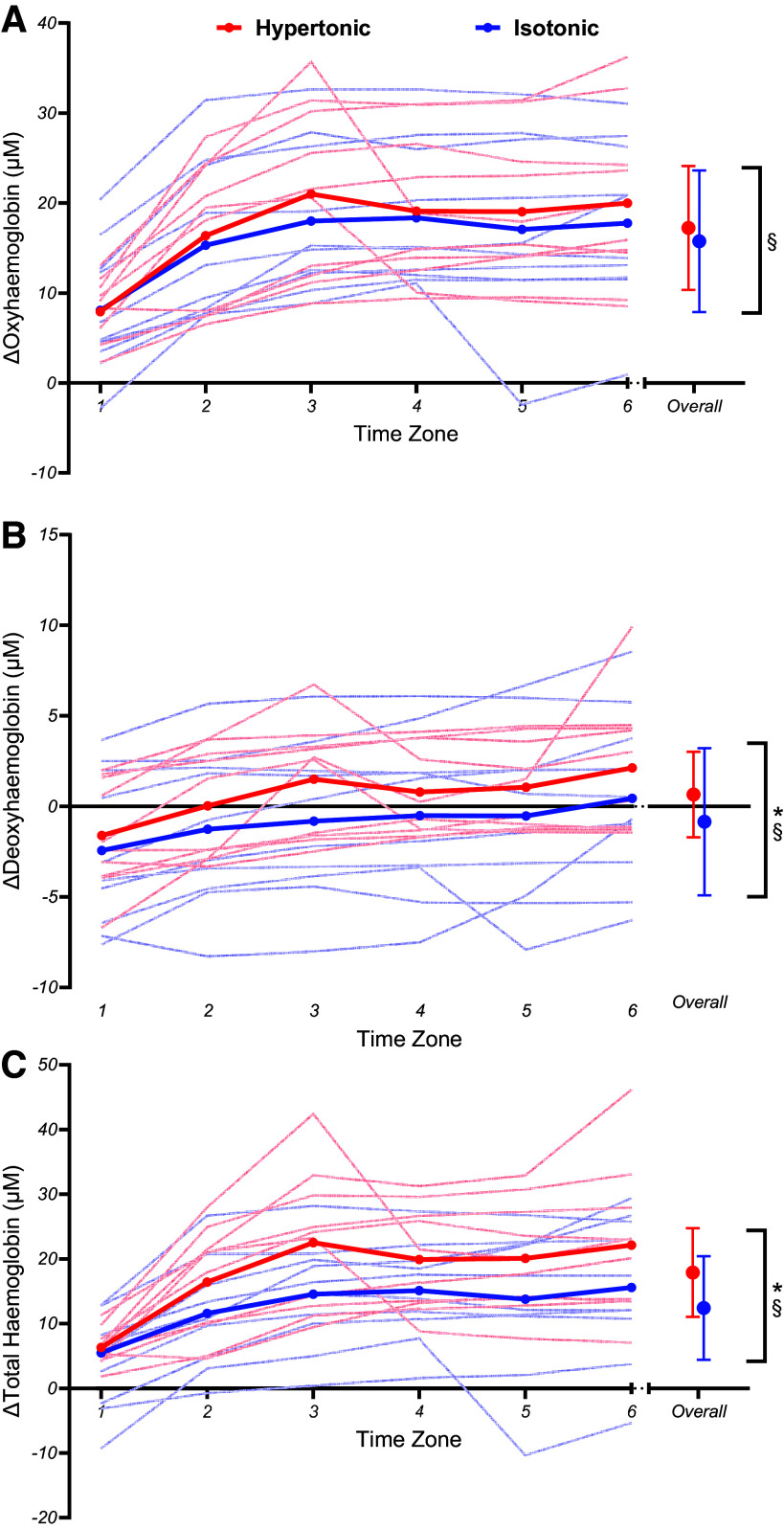
Mean group (thick line) and individual (thin lines) oxyhemoglobin (ΔO_2_Hb; *A*), deoxyhemoglobin (ΔHHb; *B*), and total hemoglobin (ΔtHb; *C*) changes during fixed perceived effort trials. Significant condition (*) and time (§) effects are illustrated. Error bars denote SDs from the mean.

A condition effect for ΔO_2_Hb was not observed (*t*_107_ = −1.71, *P* = 0.091, β = −1.48 ΔμM [−3.17,0.22]). However, a significant main effect for time (*t*_107_ = 6.81, *P* = 0.001, β = 1.72 ΔμM [1.22,2.22]) was observed for ΔO_2_Hb, as it increased over the course of the exercise in both conditions (Fig. 5*A*). The linear mixed-model regression showed no condition × time interaction for ΔO_2_Hb (*t*_107_ = −0.70, *P* = 0.486, β = −0.35 [−1.35,0.64]).

Alternatively, ΔHHb (*t*_107_ = −3.29, *P* = 0.001, β = −1.50 ΔμM [−2.40,−0.61]) and ΔtHb (*t*_107_ = −4.15, *P* = 0.001, β = −5.46 ΔμM [−8.04,−2.88]) were observed to be significantly lower in the isotonic compared to hypertonic condition (Fig. 5, *B* and *C*). Both ΔHHb (*t*_107_ = 4.04, *P* = 0.001, β = 0.54 ΔμM [0.28,0.80]) and ΔtHb (*t*_107_ = 5.65, *P* = 0.001, β = 2.18 ΔμM [1.42,2.94]) also showed a significant time-based main effect, with both increasing over the course of the exercise. However, no significant condition × time interaction was noted for ΔHHb (*t*_107_ = −0.44, *P* = 0.659, β = −0.12 [−0.64,0.41]) or ΔtHb (*t*_107_ = −0.83, *P* = 0.407, β = −0.64 [−2.15,0.87]).

Finally, no significant condition (*t*_107_ = 1.94, *P* = 0.055, β = 0.52% [−0.01,1.04]) or time (*t*_107_ = −0.58, *P* = 0.566, β = −0.04% [−0.20,0.11]) main effects were found for ΔTSI. Also, there was not a significant condition × time interaction for ΔTSI (*t*_107_ = 1.91, *P* = 0.059, β = 0.30 [−0.01,0.60]).

### Perceptual Markers

**Figure 6.**
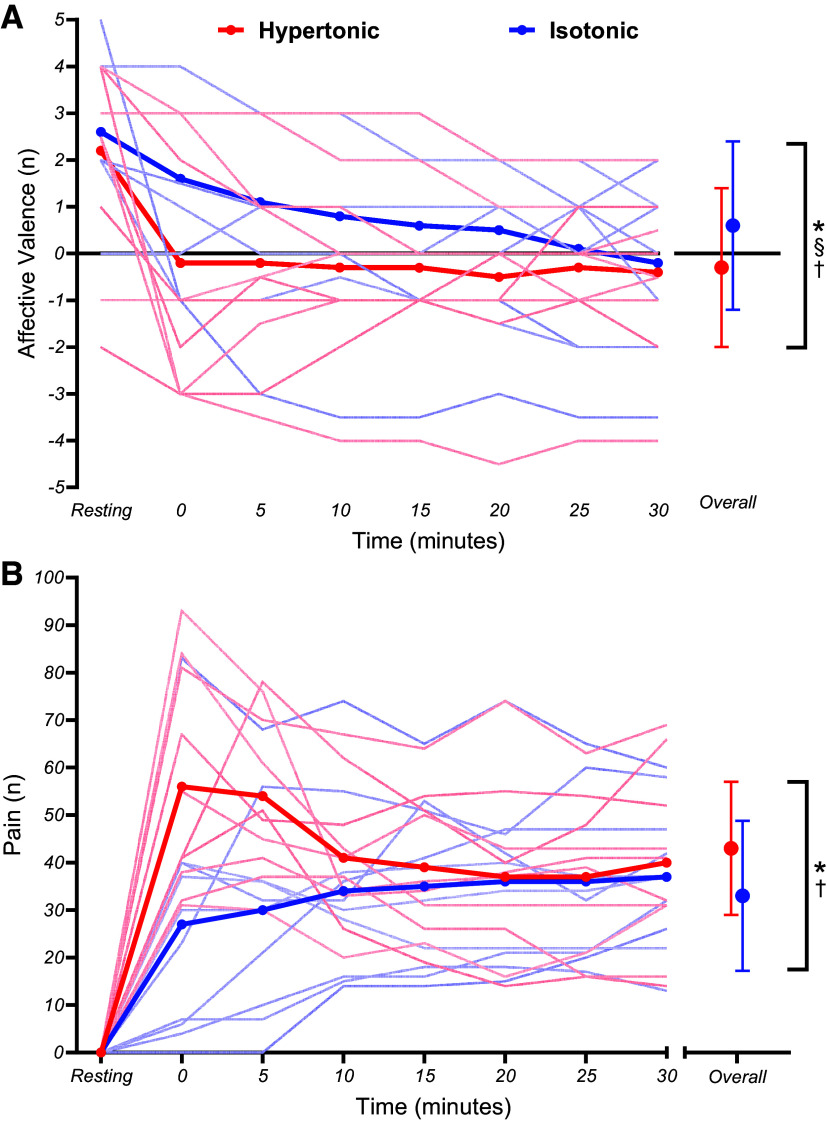
Mean group (thick line) and individual (thin lines) affective valence (*A*) and pain intensity perceptual responses (*B*) during fixed perceived effort trials. Significant condition (*), time (§), and condition × time (†) effects are illustrated. Error bars denote SDs from the mean.

Affective valence was found to be significantly lower in the hypertonic compared to isotonic condition, with a significant condition main effect (*t*_127_ = 6.12, *P* = 0.001, β = 0.93 [0.63,1.23]) as well as a significant main effect for time (*t*_127_ = −3.96, *P* = 0.001, β = −0.03 [−0.04,−0.02]). Notably, time-based changes in affective valence differed between conditions, as a linear mixed-model regression also observed a significant condition × time (*t*_127_ = −3.16, *P* = 0.002, β = 0.05 [−0.08,−0.02]) interaction. In particular, affective valence responses were more negative in earlier stages of the exercise in the hypertonic compared to isotonic condition (Fig. 6*A*).

Pain ratings were significantly higher in the hypertonic compared to isotonic condition (*t*_127_ = −5.90, *P* = 0.001, β = −9.97 [−13.28,−6.66]) (Fig. 6*B*). However, time-based main effects were not significant (*t*_127_ = −1.78, *P* = 0.077, β = −0.15 [−0.32,0.01]). Trajectories in the changes of pain ratings were significantly different between conditions, with a significant condition × time interaction (*t*_127_ = 6.00, *P* = 0.001, β = 0.95 [0.61,1.28]). In particular, pain decreased and then plateaued in the hypertonic condition and pain increased and then plateaued in the isotonic condition.

Table 1 demonstrates the dimensional quality of perceived pain during trials. Total scores for subclasses of sensory and affective domains did not demonstrate significant differences between conditions; however, a moderate effect (*d* = 0.55) in the sensory and a large effect (*d* = 0.80) in the affective domain were observed. Total scores for dimensions of evaluative (*Z* = 2.392, *P* = 0.017, *d* = 0.67), miscellaneous (*t* = 3.139, *P* = 0.012, *d* = 0.50), and pain rating index (PRI) (*Z* = 2.075, *P* = 0.038, *d* = 0.84) did demonstrate significant differences between conditions with moderate and large effect sizes.

## DISCUSSION

This study aimed to investigate the impact of elevated muscle pain through a hypertonic saline injection on the power output changes, psychophysiological state, and cerebral oxygenation variables during a fixed perceived effort exercise task. Knowledge of the changes in the power output, psychophysiological indexes, and cerebral hemodynamics also contributed to a secondary question that explored the self-regulatory strategies that were used to maintain a fixed perceived effort during conditions of pain (hypertonic) or a placebo control (isotonic).

The main finding of the present study is that the hypertonic condition elicited a significantly lower power output (by an average of 5 W) than the isotonic condition, alongside which there were no significant condition effects on any physiological variables like heart rate, V̇o_2_·kg^−1^, V̇e, breathing frequency, or blood lactate. However, differences in power output between conditions were paired with significant differences in pain intensity and quality responses, which were found to be significantly higher in the hypertonic compared to isotonic condition. Likewise, this study demonstrated significantly worse/more negative affective valence responses in the hypertonic compared to isotonic condition. Finally, there was a significantly higher change in deoxyhemoglobin levels from baseline in the hypertonic versus isotonic condition.

Findings pertaining to power output confirmed our initial hypothesis. Numerous studies have demonstrated a reduced task output (e.g., power output, force, duration on task) during painful compared to nonpainful conditions (7–12). Notably, muscle pain imposes neurophysiological alterations such as changes in corticomotor conductance of central drive (13, 14, 16) and muscle fiber recruitment (15, 17) as well as heightened psychophysiological demands such as reduced affect (19, 20). The psychophysiological consequences of pain are confirmed in this study. Namely, this study observed lower/worse affective valence responses during the hypertonic versus isotonic condition, inferring that individuals may have experienced a less hedonic experience (20) because of the pain, with further implications for their motivation to continue exercising at the same perception of effort (44), thus resulting in a negatively valenced affective response (45). Furthermore, the changes in deoxyhemoglobin from a resting baseline were significantly higher in the painful hypertonic versus less painful isotonic condition. Specifically, cerebral oxygenation measures were taken from the prefrontal cortex, which several recent studies have indicated is linked to executive function (46). Therefore, the results of this study imply that individuals during the painful, hypertonic condition engaged in more inhibitory control (a subset of executive function) to cope with pain (47–49). Notably, continued inhibitory control is closely associated with increases in effort due to enhanced activity of cortical areas (18, 20, 46, 47) associated with effort processing as well as a motivationally fatiguing effect (22). Consequently, it is expected that exercise in the presence of higher pain is more effortful than exercise without pain (1, 3, 5, 18). When the task paradigm is switched to a fixed perceived effort trial, it is expected that the task output such as power output would be lower within conditions of pain versus a control (7, 9–12). Yet some caution is warranted when considering hemodynamic responses, as there are potential confounds involving the autonomic nervous regulation of blood flow during an exercise of vigorous intensity that could impact the raw changes in oxygenation markers that were measured (34, 50).

However, it was interesting to note that there were no differences in any of the physiological/cardiorespiratory markers despite significant differences in power output, leading the authors to reject some aspects of their secondary hypotheses. Certain models of exercise regulation insist that exercise behavior is governed by afferent feedback loops that relay information through the central nervous system concerning metabolic and proprioceptive changes (51). Yet the results of this study appear in conflict with this suggestion, as physical outputs at a constant perceived intensity were not proportional to the subconscious changes in cardiorespiratory and metabolic parameters that were monitored. Alternatively, it may be worthwhile acknowledging other models [e.g., psychobiological model (11)] that claim that afferent feedback impacts exercise behavior via changes in effort perceptions. Relatedly, a recent study by Mauger et al. (52) discerned that after trained cyclists were administered tramadol (a very potent painkiller), performance in a subsequent time trial was significantly faster compared with a placebo-controlled condition. In addition, Mauger and colleagues (52) required participants to conduct a fixed-intensity cycle before their time trial and found that RPE responses were significantly lower after tramadol ingestion versus control. Therefore, some indications could be made to justify the effect that afferent feedback like nociception/pain has on the exercise performance because of its combined neurophysiological and psychophysiological influences on effort perceptions (7).

Consecutively, this study aimed to explore the self-regulatory strategies that operate during fixed perceived effort cycling in the presence of painful (hypertonic) or less painful/nonpainful (isotonic) conditions. Mainly, condition × time interactions can illustrate the differences in the changes for power output (behavioral) or cerebral hemodynamics (cognitive) self-regulation over time. Furthermore, researchers of this study were aware that a hypertonic saline procedure typically peaks at ∼3 min and dissipates within ∼5–6 min after administration (9–12, 29, 30), yet the fixed perceived effort task lasted 30 min. However, this generated another question as to whether a pain experience imposes residual effects at later stages of an exercise task, as previous studies have shown that even after a pain experience neurophysiological markers do not immediately return to baseline, perhaps because of a retained motor adaptation (15).

Results conflicted with our prior hypotheses, with no significant condition × time interactions for power output, any markers of physiological strain, or cerebral oxygenation parameters. Figure 2 illustrates that both conditions exhibited an expected decrease in power output (31) but the rate at which power output decreased was unaffected. Meanwhile, markers of physiological strain (Fig. 3) indexed a plateau that would be expected for certain markers like breathing frequency during fixed perceived effort exercise (53). Similarly, changes in oxy-, deoxy-, and total hemoglobin over the course of the fixed perceived effort bouts were not significantly different between conditions (Fig. 5). Instead, the only significant condition × time interactions that were observed related to the pain intensity and affective valence responses (Fig. 6). Naturally, differences in pain intensity responses were expected, as the hypertonic condition evoked higher perceptions of pain compared with the isotonic condition at the start of the exercise whereas the progressive engagement in exercise caused naturally occurring muscle pain to reach similar levels in the latter stages of the task (8). Second, the affective valence responses demonstrated that the painful hypertonic saline conditions caused affect to become more negative/worse much sooner, whereas the isotonic condition caused affect to become negative at a much steadier rate. However, it is interesting that this difference in affective valence did not instigate any differences in self-regulatory behavior (i.e., changes in power output) as some may expect (54).

Consequently, two main conclusions can be drawn about the self-regulation of perceived effort during conditions of pain versus less painful/nonpainful conditions. First, it appears that pain does prompt a difference in task outputs at a set perception of effort, as shown by the condition effects for power output and cerebral oxygenation markers. A second conclusion is that the pain ratings and power output data indicate that pain does affect the perception of effort and associated outputs but only when it is experienced. Alternatively, pain does not seem to demonstrate any residual effects that impact exercise behavior at a later stage of a task when elevated muscle pain has dissipated. To illustrate, there were no significant condition × time interactions, suggesting that although higher pain ratings at the start of the exercise may be indicative of increased engagement in inhibitory control, this may not be an enduring effect on exercise behavior as prior resource models of self-regulation would suggest (5).

### Limitations and Future Research

Some aspects of this study’s methodological approach could be adapted in future studies to understand more about the effect of pain on perceived effort and the subsequent self-regulation of exercise behavior. One note is that this study did not control for the volume of the saline bolus in accordance with muscle mass. Instead, all participants were administered a bolus of 1 mL of saline. As a result, those with lower vastus lateralis mass may have experienced a higher intensity of pain versus those with greater muscle mass. Observation of the pain data (Fig. 6*B*) does show a varied response to the hypertonic saline when it was most potent (*minutes 0* and *5*). As a result, this may in part contribute to the slightly larger variances in power output (95% CI = 0–9 W lower in the hypertonic vs. isotonic condition over 30 min).

Another aspect of the varied power output response may have been due to the duration of the fixed perceived effort task. As noted above, although the 30-min task duration afforded researchers the opportunity to observe any potential residual effects of pain on exercise behavior, the differences in later stages of the task were negligible (2–4 W), thus skewing the observed effects and increasing the likelihood of a type II error. However, the results did show an average difference of 10–25 W at *minutes 0–5* while the pain intensity was higher due to the hypertonic saline (Fig. 2), a result that is both statistically as well as physiologically meaningful. In context, individuals experiencing high levels of pain are likely to conduct a given task at a much slower rate, with potentially inferior performance (7–12, 20–22, 29). In addition, an overall average (i.e., the entire 30-min group mean) exhibited a ∼5 W lower power output in the painful versus isotonic condition. Although this result may not be entirely meaningful for everyday situations, it is still statistically significant and could still be considered relevant to elite sporting populations. For instance, RPE responses ∼15 (“hard”) are commonplace at the initial phases of a prolonged time trial (55). Therefore, if a competitor can gain an initial advantage due to a higher power output at the start of a race-type situation because they are free from any existing pain, this is contextually meaningful (55).

Finally, although this study aims to incorporate the best practice for fNIRS measurement (34), some aspects of data collection were not viable. For example, Pinti et al. (34) suggest that the additional use of short-separation channels to obtain fNIRS data may allow a better interpretation of fNIRS neuroimaging data when analyzed with linear mixed-model regression like those used in this study. In addition, short-separation channels can detect additional noise from extracerebral signals (e.g., cardiac cycles), which can subsequently factor into the analysis of data to eradicate confounds such as systemic interference as a consequence of the exercise. However, as this study was concerned with oxy/deoxyhemoglobin changes at the prefrontal cortex, long-separation, single channels were used because of the need for penetration to deeper tissues (e.g., cerebral vs. muscle fNIRS). Although filters identical to previous studies in the area were used to eradicate potential noise and confounds (33, 35–37), some caution is warranted in the interpretation of fNIRS data.

In accordance with these shortcomings, future research may wish to control for the volume of saline that is applied according to muscle mass. Furthermore, the duration of a task could be curtailed to fit the expected time for which saline procedures remain effective (∼5–6 min). Beyond this, other suggestions for future research could involve other markers of cognitive effort. Although several studies have hinted toward cerebral oxygenation markers as being indicative of cognitive effort (47–50), other methods such as preejection period and eye tracking (e.g., measurement of pupil diameter and/or variability in fixation locations) are potentially effective at measuring cognitive load/effort through another physiological approach (56, 57). Characteristically, exercise tasks impose physical and cognitive demands, but little is known about the ways in which individuals choose between applying physical or cognitive effort (2, 4). Therefore, future research could explore this area, as it could shed light into how psychophysiological constructs like pain and effort are regulated and influence exercise behaviors and performance.

### Conclusions

The present study aimed to investigate the impact of elevated pain perceptions through a hypertonic saline injection on power output and psychophysiological state during a fixed perceived effort task. It was observed that the painful hypertonic condition caused a significantly lower power output, a greater increase in deoxyhemoglobin compared with rest, and a lower/worse affective response compared with a placebo-controlled isotonic condition. However, there were no differences in any markers of physiological strain between conditions. Therefore, it may be that the regulation of exercise behavior like power output is not directly related to physiological parameters but may operate via the perception of effort.

In addition, the present study also aimed to investigate the changes in power output (behavioral) and cerebral hemodynamics (cognitive) as indicators of the self-regulatory strategies that were used to maintain a fixed perceived effort during conditions of pain (hypertonic) or a control (isotonic). However, no significant condition × time interactions were detected for power output, physiological, or cerebral oxygenation markers. Therefore, it was concluded that pain impacts the self-regulation of fixed perceived effort exercise, as differences in power output mainly occurred when pain ratings were higher after hypertonic versus isotonic saline administration.

An emphasis in our discussion highlights the potential impacts our approach may have for the conclusions on pain’s effect of perceived effort and subsequent exercise behavior. Furthermore, we propose potential avenues for future research to account for the shortcomings of our approach and other ways that physical and cognitive effort contributions operate during self-regulated exercise tasks.

## DATA AVAILABILITY

Data are available upon request from the corresponding author in raw and analyzed forms. Data can be seen at https://www.doi.org/10.17605/OSF.IO/3JVU2, with some additional materials related to the study provided.

## SUPPLEMENTAL MATERIALS

10.17605/OSF.IO/3JVU2 Supplemental materials: https://www.doi.org/10.17605/OSF.IO/3JVU2.

## DISCLAIMERS

No disclaimers are required as part of this work.

## DISCLOSURES

No conflicts of interest, financial or otherwise, are declared by the author(s). For the purpose of open access, the author has applied a Creative Commons Attribution (CC BY) license to any Author Accepted Manuscript version arising from this submission.

## AUTHOR CONTRIBUTIONS

C.A.O., C.L.F., and A.R.M. conceived and designed research; C.A.O., R.N., S.A.S., and A.R.M. performed experiments; C.A.O. analyzed data; C.A.O. interpreted results of experiments; C.A.O. prepared figures; C.A.O. drafted manuscript; C.A.O., R.N., S.A.S., C.L.F., and A.R.M. edited and revised manuscript; C.A.O., R.N., S.A.S., C.L.F., and A.R.M. approved final version of manuscript.
